# Association of Cyberbullying Experiences and Perpetration With Suicidality in Early Adolescence

**DOI:** 10.1001/jamanetworkopen.2022.18746

**Published:** 2022-06-27

**Authors:** Shay Arnon, Anat Brunstein Klomek, Elina Visoki, Tyler M. Moore, Stirling T. Argabright, Grace E. DiDomenico, Tami D. Benton, Ran Barzilay

**Affiliations:** 1 Baruch Ivcher School of Psychology, Reichman University, Herzliya, Israel; 2 Department of Child and Adolescent Psychiatry and Behavioral Science, Children’s Hospital of Philadelphia, Philadelphia, Pennsylvania; 3 Lifespan Brain Institute of Children’s Hospital of Philadelphia and Penn Medicine, Philadelphia, Pennsylvania; 4 Department of Psychiatry, Perelman School of Medicine, University of Pennsylvania, Philadelphia

## Abstract

**Importance:**

Adolescent suicidality (ie, suicidal ideation or attempts) is a major public health concern. Cyberbullying experiences and perpetration have become increasingly prevalent and are associated with mental health burden, but their roles as independent suicidality risk factors remain unclear. Data are needed to clarify their contribution to teen suicidality to inform suicide prevention efforts.

**Objective:**

To examine whether cyberbullying experiences and perpetration are distinct stressors divergent from other forms of peer aggression experiences in their association with suicidality in early adolescence.

**Design, Setting, and Participants:**

This cross-sectional analysis used data collected between July 2018 and January 2021 from the Adolescent Brain Cognitive Development (ABCD) study, a large, diverse sample of US children aged 10 to 13 years.

**Exposures:**

Youth reports of cyberbullying experiences or perpetration.

**Main Outcomes and Measures:**

The main outcome was youth-reported suicidality (past or present, as reported in the ABCD 2-year follow-up assessment). Covariates included demographics, established environmental risk and protective factors for youth suicidality, psychopathology, and experiences or perpetration of offline peer aggression.

**Results:**

A total of 10 414 ABCD participants were included in this study. Participants had a mean (SD) age of 12.0 (0.7) years and 4962 (47.6%) were female; 796 (7.6%) endorsed suicidality. A total of 930 (8.9%) reported experiencing cyberbullying and 96 (0.9%) reported perpetrating cyberbullying. Of the perpetrators, 66 (69.0%) also endorsed experiencing cyberbullying. Controlling for demographics, experiencing cyberbullying was associated with suicidality (odds ratio [OR], 4.2 [95% CI, 3.5-5.1]; *P* < .001), whereas perpetrating cyberbullying was not (OR, 1.3 [95% CI, 0.8-2.3]; *P* = .30). Experiencing cyberbullying remained associated with suicidality when accounting for negative life events, family conflict, parental monitoring, school environment, and racial and ethnic discrimination (OR, 2.5 [95% CI, 2.0-3.0]; *P* < .001) and when further covarying for internalizing and externalizing psychopathology (OR, 1.8 [95% CI, 1.4-2.4]; *P* < .001). Both being a target and being a perpetrator of offline peer aggression were associated with suicidality (OR, 1.5 [95% CI, 1.1-2.0] for both), controlling for all covariates described earlier. Cyberbullying experiences remained associated with suicidality (OR, 1.7 [95% CI, 1.3-2.2]; *P* < .001, controlling for all covariates) when included with offline peer aggression experiences and perpetration.

**Conclusions and Relevance:**

In this cross-sectional study, experiencing—but not perpetrating—cyberbullying was associated with suicidality in early adolescence. This association was significant over and above other suicidality risk factors, including offline peer aggression experiences or perpetration. These findings can inform adolescent suicide prevention strategies, and they suggest that clinicians and educational staff working with this population should routinely evaluate for adolescents’ experience with cyberbullying.

## Key Points

**Question:**

Is involvement in cyberbullying independently associated with suicidality (ideation or attempts) in early adolescence?

**Findings:**

In this cross-sectional analysis of 10 414 US adolescents aged 10 to 13 years, experiencing cyberbullying was associated with suicidality but perpetrating cyberbullying was not. The association with suicidality remained for targets of cyberbullying even when accounting for multiple confounders, including experiences or perpetration of offline peer aggression.

**Meaning:**

This study suggests that identification of cyberbullying experiences can assist clinicians in adolescent suicide risk stratification and can inform youth suicide prevention strategies.

## Teaser

This cross-sectional study examines the association of suicidality in early adolescence with experiences or perpetration of cyberbullying among US youths aged 10 to 13 years in the Adolescent Brain Cognitive Development study.

## Introduction

Suicidality (suicidal ideation or suicide attempts) among adolescents is a major public health concern,^1^ with suicide being the second leading cause of death among US adolescents and young adults aged 10 to 24 years.^2^ The etiology of youth suicidality is not fully understood but is known to be influenced by distal and proximal environmental stressors,^3,4^ which are especially critical during childhood and adolescence.^5^ Experiences of peer bullying and peer aggression are major stressors and established suicidality risk factors among youths.^6,7,8,9^ Most contemporary bullying is conducted online (ie, cyberbullying) using technological platforms such as smartphones and the internet (eg, text messages, social media).^10,11^ This trend has further increased as a result of social changes attributable to the COVID-19 pandemic.^12,13^ In an era of increasing online relationships and interactions^14,15,16^ as well as increased mental health^17^ and suicidality burden among youths,^18,19,20,21^ more large-scale data are needed to clarify the role of cyberbullying involvement in youth suicidality.

Youths involved in cyberbullying have greater psychopathology^22,23^ and experience more suicidality.^24,25,26^ Both targets and perpetrators (although to a lesser extent) of cyberbullying are at increased risk for suicidality compared with their peers not involved in cyberbullying.^25^ Notably, some evidence suggests that youths who both experience and perpetrate cyberbullying are most at risk.^27,28,29,30,31^ The literature is ambiguous regarding whether cyberbullying involvement is a distinct suicidality risk factor independent of experiences or perpetration of offline peer aggression.^24,31,32^ Some researchers highlight the overlap of experiences or perpetration of offline peer aggression and cyberbullying experiences, claiming that cyberbullying is a subtype of offline peer aggression with limited distinguishable effects on youth mental health.^33,34,35^ More research is needed to better understand whether cyberbullying experiences as well as different cyberbullying roles are associated with suicidality over and above other risk factors. Such data are pivotal for informing youth suicide prevention efforts, especially in the post–COVID-19 era, considering the pandemic’s toll on mental health among youths.^36,37,38^

For this cross-sectional study, we leveraged data from a large, diverse sample of US adolescents (aged 10-13 years) from the Adolescent Brain Cognitive Development (ABCD) study.^39^ We aimed to (1) determine the prevalence and overlap of cyberbullying experiences (for targets and perpetrators); (2) evaluate the specific associations of cyberbullying experiences with suicidality, over and above multiple other environmental risk factors (ie, the exposome^40,41^); and (3) evaluate whether cyberbullying experiences are associated with suicidality, over and above offline peer aggression experiences and perpetration.

## Methods

### Participants

The ABCD study comprised 11 878 children ascertained at age 9 to 10 years at baseline through US school systems.^31^ Participants were enrolled at 21 sites, with the catchment area encompassing more than 20.0% of the US population in this age group. For this cross-sectional observation study, we included data from ABCD data release 4.0, the first release of the cyberbullying data for the entire cohort.^42^ Data for all included measures were collected at the 2-year follow-up assessment for 10 414 participants between July 2018 and January 2021 with the exception of demographic data that were collected at either 1-year follow-up (parent education) or baseline (sex, race and ethnicity) assessment. All participants provided assent, and parents or caregivers provided written informed consent. The ABCD protocol was approved by the University of California, San Diego Institutional Review Board and was deemed exempt from full review by the University of Pennsylvania Institutional Review Board. This study followed the Strengthening the Reporting of Observational Studies in Epidemiology (STROBE) reporting guideline.^43^

### Exposures

Cyberbullying experiences were assessed with the ABCD Cyber Bully Questionnaire, which defined cyberbullying as “purposefully trying to harm another person or be mean to them online, in texts or group texts, or on social media (like Instagram or Snapchat).”^44^ Our main exposures were based on 2 binary (yes or no) questions regarding past experiences of being either a target (“cybervictim”) or a perpetrator (“cyberbully”) of cyberbullying (measures cybb_phenx_harm and cybb_phenx_harm2, respectively). In separate models, we considered the power imbalance within the cyberbullying relationship and the chronicity of cyberbullying exposures in the past year.

Offline peer aggression experiences and perpetration were assessed with the Peer Experiences Questionnaire.^45^ Given that this questionnaire does not inquire about a power imbalance between the perpetrator and the target, which is essential to the construct of bullying,^46^ this study refers to these exposures as offline peer aggression experiences and perpetration throughout.

The Peer Experiences Questionnaire probed 3 distinct domains of peer experiences: overt aggression (eg, threatening, hitting), relational aggression (eg, not inviting or leaving someone out), and reputational aggression (eg, spreading rumors, gossiping that hurt one’s reputation). Participants were asked whether they were a target (“offline peer victim”) or a perpetrator (“offline peer aggressor”) of offline peer experiences within these 3 domains (measures peq_ss_overt_victim, peq_ss_relational_victim, and peq_ss_reputation_victim and peq_ss_overt_aggression, peq_ss_relational_aggs, ands peq_ss_reputation_aggs, respectively). Each domain score was calculated from the sum of 3 relevant questions measured on a Likert scale from 1 (“never”) to 5 (“a few times a week”); thus, each domain score was measured on a scale from 3 to 15.

Experiences and perpetration of offline peer aggression correlated across the overt, relational, and reputational domains (Pearson *r* = 0.2-0.55; all *P* < .001) (eFigure in the Supplement). We created summary measures of experiences and perpetration of offline peer aggression by summing their individual domain scores; thus, each measure was scored on a scale from 9 to 45. In the main analyses, we dichotomized each summary measure based on whether participants scored in the top decile of endorsed experiences, which we refer to herein as being a high offline peer aggression target and a high offline peer aggression perpetrator.

### Outcome Measures

Past and current suicidal ideation and suicide attempts were evaluated using the self-report Kiddie Schedule for Affective Disorders and Schizophrenia for the *Diagnostic and Statistical Manual of Mental Disorders* (Fifth Edition).^47^ We combined suicidal ideation and attempts, consistent with previous analyses, because the proportion of suicide attempts was low and because we aimed to mitigate type I error risk caused by multiple testing.^48,49,50,51^ In the main analyses, past and current suicidal ideation and attempts were collapsed into a single binary measure termed “suicidality.” In sensitivity analyses, we tested associations of exposures with suicidal ideation and suicide attempts separately. Because previous reports (including from the ABCD study) showed poor youth-caregiver agreement on suicidality,^52,53^ we focused on youth reports in the main analyses.

### Covariates

The models included age, sex, race (Black or White), ethnicity (Hispanic), and parent education. Race and ethnicity data were self-reported and were collected owing to disparities in youth suicidality and specifically in the ABCD study.^49^ To address confounding effects of other variables previously linked to childhood suicidality in the ABCD study,^48,49,51,53^ we ran models covarying for family conflict, parental supervision, school environment, negative life events, and experiences of racial and ethnic discrimination. To address the confounding effect of comorbid (nonsuicidality) psychopathology, we included measures representing internal and external psychopathology derived from youth reports (Brief Problem Monitor^54^) and parent reports (Child Behavioral Checklist^55^), respectively.

### Statistical Analysis

The statistical analysis plan and hypotheses were preregistered on the Open Science Framework in October 2021. Statistical analyses were conducted from December 1, 2021, to January 31, 2022, following ABCD data release 4.0.

Means (SDs) and frequencies are reported for descriptive purposes. Analysis of variance or χ^2^ tests were used for univariate comparisons, as appropriate. We used 2-tailed tests for all statistical models and set statistical significance at *P* = .05. We used listwise deletion for participants with missing data (0.5% for cyberbullying exposures and 1.0% for suicidality). For data analyses, we used IBM SPSS, version 26.0, and R, version 4.1.0 (R Group for Statistical Computing).

To investigate associations of cyberbullying experiences and suicidality, we estimated binary logistic regression models in which the dependent variable was youth-reported suicidality and the independent variables were experiencing cyberbullying and perpetrating cyberbullying (both yes or no). Covariates included demographics (model 1; age, sex, race and ethnicity, and parents’ education), additional environmental factors previously associated with suicidality in the ABCD study (model 2; negative life events, parental monitoring, school protective factors, family conflict, and racial and ethnic discrimination), and additional measures of psychopathology (model 3; youth-report Brief Problem Monitor and parent-report Child Behavioral Checklist, internalizing and externalizing *t* scores). We tested the interaction of experiencing cyberbullying and perpetrating cyberbullying in a separate model that included the product of these 2 exposures.

To investigate associations of suicidality and experiences or perpetration of offline peer aggression, we estimated similar binary logistic regression models as described earlier but instead of cyberbullying experiences, we included the 2 summary measures high offline peer aggression target and high offline peer aggression perpetrator (each dichotomized as above the top decile score vs the rest of the sample).

To address the question of whether experiencing cyberbullying is associated with suicidality over and above offline peer aggression experiences and perpetration, we conducted binary logistic regression models as described earlier but including the measures of being a cyberbullying target, high offline peer aggression target, and high offline peer aggression perpetrator in the same model.

We also conducted several sensitivity analyses as follows. To account for the potential influence of our choice of exposure variables, we ran the main models using different measures of cyberbullying that consider power imbalance and frequency over the past year. We also ran models of offline peer experiences using the overt, relational, and reputational aggression domains separately.

To account for site and family-relatedness effects on the association of cyberbullying experiences and suicidality, we estimated a multilevel logistic regression model using the Mplus robust maximum likelihood estimator,^56^ consistent with previous ABCD research.^57^ Finally, we tested the association of cyberbullying experiences with suicidal ideation and suicide attempts in separate analyses.

## Results

### Cyberbullying Prevalence

A total of 10 414 youths participated in the 2-year follow-up ABCD assessment. The mean (SD) participant age was 12.0 (0.7) years; 4962 (47.6%) were female and 5452 (52.4%) were male. A total of 2057 participants (19.8%) were Black, 2086 were Hispanic (20.0%), and 7894 (75.8%) were White. There were 796 participants (7.6%) who endorsed suicidality (785 [7.5%] reported suicidal ideation and 152 [1.5%] endorsed attempt). In addition, 930 participants (8.9%) reported being a target of cyberbullying and 96 (0.9%) reported being a perpetrator of cyberbullying. Female and Black participants were more likely than male participants (488 [9.8%] vs 442 [8.1%]; *P* = .002) and participants of any race or ethnicity other than Black (215 [10.5%] vs 715 [8.6%]; *P* = .005) to experience cyberbullying, respectively. Male and Black participants were more likely than female participants (1.1% vs 0.7%; *P* = .04) and participants of any race or ethnicity other than Black (1.6% vs 0.8%; *P* < .001) to be perpetrators of cyberbullying, respectively. We found no differences in age or ethnicity among youths who experienced or perpetrated cyberbullying compared with their peers not involved in cyberbullying (Table 1).

**Table 1.** Sociodemographic and Clinical Characteristics of the Study Participants

Characteristic	ABCD participants experiencing cyberbullying, No. (%)					*P* value^b^
	Total (N = 10 414)^a^	Target only (n = 857)	Perpetrator only (n = 30)	Both target and perpetrator (n = 66)	No experiences (n = 9393)	
Age, mean (SD), y	12.0 (0.7)	12.0 (0.7)	12.1 (0.8)	12.1 (0.7)	12.0 (0.7)	.33
Parent education, mean (SD), y	16.6 (2.6)	16.3 (2.5)	15.8 (2.5)	15.8 (2.4)	16.6 (2.6)	<.001
Sex						
Female	4962 (47.6)	461 (53.8)	10 (33.3)	26 (39.4)	4435 (47.2)	<.001
Male	5452 (52.4)	396 (46.2)	20 (66.7)	40 (60.6)	4958 (52.8)	
Race						
Black	2057 (19.8)	194 (22.6)	15 (50.0)	18 (27.3)	1804 (19.2)	<.001
White	7894 (75.8)	641 (74.8)	12 (40.0)	41 (62.1)	7156 (76.2)	<.001
Hispanic ethnicity	2086 (20.0)	182 (21.2)	8 (26.7)	10 (15.2)	1875 (20.0)	.49
Suicidality	796 (7.6)	192 (22.4)	4 (13.3)	16 (24.2)	573 (6.1)	<.001
Suicidal ideation	785 (7.5)	190 (22.2)	4 (13.3)	16 (24.2)	564 (6.0)	<.001
Suicide attempt	152 (1.5)	49 (5.7)	0 (0.0)	5 (7.6)	94 (1.0)	<.001

Abbreviation: ABCD, Adolescent Brain Cognitive Development.

^a^ The missing data rate was 0.5% for both targets and perpetrators of cyberbullying. Only participants with available data for both cyberbullying experiences and perpetration were included here; thus, the total missingness of cyberexposure data was 0.7%. For suicidality measures, the missing data rate was 1.0% among the 10 414 participants at the 2-year follow-up ABCD study assessment.

^b^ Analysis of variance and χ^2^ test comparisons were used for continuous and binary measures, respectively.

Of the 96 perpetrators of cyberbullying, 66 (69.0%) also endorsed experiencing cyberbullying. Given the literature on offline peer aggression suggesting that youths who both experience and perpetrate cyberbullying have a greater risk for suicidality,^6,58,59,60^ we first evaluated whether there are differences in suicidality rates among youth targets of cyberbullying, perpetrators of cyberbullying, and those who engage in both. We found that targets of cyberbullying endorsed more suicidality regardless of whether they were a target only (857 [22.4%]) or both a target and a perpetrator (66 [24.2%]) compared with their peers not involved in cyberbullying (573 [6.1%]) (Table 1). Only 30 participants (0.3%) in the cohort endorsed perpetrating but not experiencing cyberbullying, with 4 (13.3%) reporting suicidality.

### Association of Cyberbullying Experiences With Suicidality

We next sought to delineate the association of the experiences of targets and perpetrators of cyberbullying and suicidality (Table 2). Controlling for demographics, experiencing cyberbullying was associated with suicidality with a medium effect size (odds ratio [OR], 4.2 [95% CI, 3.5-5.1]; *P* < .001), whereas perpetrating cyberbullying was not (OR, 1.3 [95% CI, 0.8-2.3]; *P* = .30). There was no interaction of experiencing and perpetrating cyberbullying in association with suicidality. Experiencing cyberbullying remained associated with suicidality when accounting for multiple environmental risk and protective factors previously linked to suicidality in the ABCD study (OR, 2.5 [95% CI, 2.0-3.0]; *P* < .001) and when further covarying for psychopathology (OR, 1.8 [95% CI, 1.4-2.4]; *P* < .001). Perpetration of cyberbullying was not associated with suicidality in any of the models.

**Table 2.** Association of Experiences and Perpetration of Cyberbullying With Suicidality in the Adolescent Brain Cognitive Development Study^a^

Characteristic	Model 1^b^		Model 2^c^		Model 3^d^	
	OR (95% CI)	*P* value	OR (95% CI)	*P* value	OR (95% CI)	*P* value
Target of cyberbullying	4.2 (3.5-5.1)	<.001	2.5 (2.0-3.0)	<.001	1.8 (1.4-2.4)	<.001
Perpetrator of cyberbullying	1.3 (0.8-2.3)	.30	0.7 (0.4-1.3)	.25	0.6 (0.3-1.4)	.26
Negative life events	NA	NA	1.2 (1.1-1.2)	<.001	1.1 (1.1-1.1)	<.001
Parental monitoring	NA	NA	0.6 (0.5-0.7)	<.001	0.7 (0.6-0.9)	.002
School protective factors	NA	NA	0.9 (0.9-1.0)	<.001	1.0 (1.0-1.0)	.02
Family conflict	NA	NA	1.2 (1.1-1.2)	<.001	1.1 (1.0-1.2)	.001
Racial or ethnic discrimination	NA	NA	1.4 (1.2-1.7)	<.001	1.0 (0.7-1.3)	.78
BPM questionnaire score						
Internalizing	NA	NA	NA	NA	1.1 (1.1-1.1)	<.001
Externalizing	NA	NA	NA	NA	1.0 (1.0-1.0)	.02
CBCL questionnaire score						
Internalizing	NA	NA	NA	NA	1.0 (1.0-1.0)	.03
Externalizing	NA	NA	NA	NA	1.0 (1.0-1.0)	.002

Abbreviations: BPM, Brief Problem Monitor; CBCL, Child Behavioral Checklist; NA, not applicable; OR, odds ratio.

^a^ Binary logistic regression models were used, with cyberbullying experiences and cyberbullying perpetration as the independent variables and suicidality as the dependent variable.

^b^ Model 1 covaries for age, sex, race (Black or White), ethnicity (Hispanic), and parent education.

^c^ Model 2 covaries as model 1 plus negative life events, parental monitoring, school protective factors, family conflict, and scores on a 7-item racial and ethnic discrimination measure (Adolescent Brain Cognitive Development study variable name dim_y_ss_mean).

^d^ Model 3 includes all covariates from model 2 plus psychopathology measures (parent and child reports of internalizing and externalizing symptoms on the CBCL and BPM questionnaires, respectively).

### Association of Offline Experiences and Perpetration of Peer Aggression With Suicidality

Both experiences and perpetration of offline peer aggression were associated with suicidality (Table 3), with high offline peer aggression target having a medium effect size (OR, 3.6 [95% CI, 2.9-4.4]) and high offline peer aggression perpetrator having a similar effect size (OR, 2.8 [95% CI, 2.3-3.5], with multivariable logistic regression models controlling for demographics; *P* < .001). Associations of offline experiences and perpetration of peer aggression with suicidality remained significant when covarying for environmental factors (OR, 2.1 [95% CI, 1.7-2.7] and 1.7 [95% CI, 1.4-2.2], respectively; *P* < .001) and when further accounting for psychopathology (both OR, 1.5 [95% CI, 1.1-2.0]; *P* = .005 and .01, respectively).

**Table 3.** Association of Experiences and Perpetration of Offline Peer Aggression With Suicidality in the Adolescent Brain Cognitive Development Study^a^

Characteristic	Model 1^b^		Model 2^c^		Model 3^d^	
	OR (95% CI)	*P* value	OR (95% CI)	*P* value	OR (95% CI)	*P* value
Target of offline peer aggression^e^	3.6 (2.9-4.4)	<.001	2.1 (1.7-2.7)	<.001	1.5 (1.1-2.0)	.005
Perpetrator of offline peer aggression^e^	2.8 (2.3-3.5)	<.001	1.7 (1.4-2.2)	<.001	1.5 (1.1-2.0)	.01
Negative life events	NA	NA	1.2 (1.1-1.2)	<.001	1.1 (1.0-1.1)	<.001
Parental monitoring	NA	NA	0.6 (0.5-0.7)	<.001	0.7 (0.6-0.9)	.002
School protective factors	NA	NA	0.9 (0.9-1.0)	<.001	1.0 (1.0-1.0)	.03
Family conflict	NA	NA	1.1 (1.1-1.2)	<.001	1.1 (1.0-1.1)	.002
Racial and ethnic discrimination	NA	NA	1.3 (1.1-1.6)	.009	0.9 (0.7-1.2)	.62
BPM questionnaire score						
Internalizing	NA	NA	NA	NA	1.1 (1.1-1.1)	<.001
Externalizing	NA	NA	NA	NA	1.0 (1.0-1.0)	.10
CBCL questionnaire score						
Internalizing	NA	NA	NA	NA	1.0 (1.0-1.0)	.02
Externalizing	NA	NA	NA	NA	1.0 (1.0-1.0)	.002

Abbreviations: BPM, Brief Problem Monitor; CBCL, Child Behavioral Checklist; NA, not applicable; OR, odds ratio.

^a^ Binary logistic regression models were used, with experiences or perpetration of offline peer aggression as the independent variables and suicidality as the dependent variable.

^b^ Model 1 covaries for age, sex, race (Black or White), ethnicity (Hispanic), and parent education.

^c^ Model 2 covaries as model 1 plus negative life events, parental monitoring, school protective factors, family conflict, and scores on a 7-item racial and ethnic discrimination measure (Adolescent Brain Cognitive Development study variable name dim_y_ss_mean).

^d^ Model 3 includes all covariates from model 2 plus psychopathology measures (parent and child reports of internalizing and externalizing symptoms on the CBCL and BPM questionnaires, respectively).

^e^ Experiences and perpetration of offline peer aggression were defined as scoring in the top decile range on the summary measure of each peer aggression experience measures (overt aggression, relational aggression, and reputational aggression).

### Association of Cyberbullying With Suicidality While Accounting for Offline Experiences and Perpetration of Peer Aggression

There was moderate overlap between experiencing cyberbullying and involvement in offline peer aggression (eTable 1 in the Supplement). For example, 262 (28.2%) and 182 (19.6%) of the cyberbullying targets reported being high offline peer aggression targets or perpetrators, respectively.

Being a target of cyberbullying was associated with suicidality over and above experiences of perpetration of offline peer aggression (Figure). This association remained significant when accounting for demographics (OR, 2.9 [95% CI, 2.4-3.6]; *P* < .001) and environmental factors (OR, 2.1 [95% CI, 1.7-2.6]; *P* < .001) and when further covarying for psychopathology (OR, 1.7 [95% CI, 1.3-2.2], *P* < .001) (Table 4).

**Figure.**
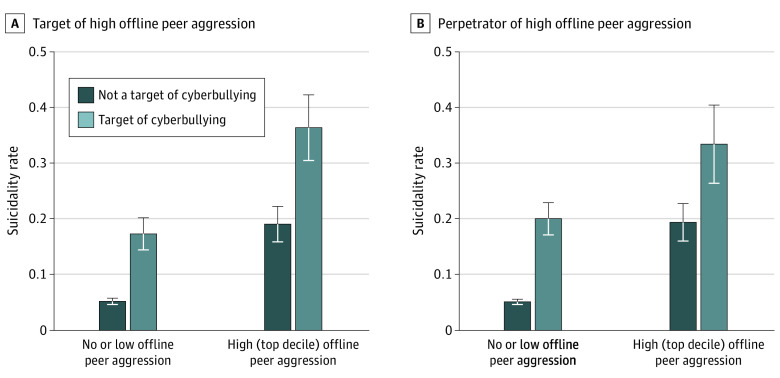
Association of Being a Target of Cyberbullying With Suicidality in Youths Who Are Targets or Perpetrators of High Levels of Offline Peer Aggression Measures of experiences (A) or perpetration (B) of offline peer aggression were created by summing scores across the 3 individual domains of peer experiences assessed in the Adolescent Brain Cognitive Development study (overt aggression, relational aggression, and reputational aggression). Summary measures were dichotomized based on whether participants scored in the top decile (ie, high) of experiencing or perpetrating offline peer aggression.

**Table 4.** Association of Cyberbullying Experiences With Suicidality in the Adolescent Brain Cognitive Development Study, Accounting for Experiences or Perpetration of Offline Peer Aggression^a^

Characteristic	Model 1^b^		Model 2^c^		Model 3^d^	
	OR (95% CI)	*P* value	OR (95% CI)	*P* value	OR (95% CI)	*P* value
Target of cyberbullying	2.9 (2.4-3.6)	<.001	2.1 (1.7-2.6)	<.001	1.7 (1.3-2.2)	<.001
Target of offline peer aggression^e^	2.8 (2.3-3.5)	<.001	1.9 (1.5-2.4)	<.001	1.4 (1.1-1.9)	.01
Perpetrator of offline peer aggression^e^	2.5 (2.0-3.1)	<.001	1.6 (1.3-2.0)	<.001	1.4 (1.0-1.9)	.04
Negative life events	NA	NA	1.1 (1.1-1.2)	<.001	1.1 (1.0-1.1)	<.001
Parental monitoring	NA	NA	0.6 (0.5-0.7)	<.001	0.7 (0.6-0.9)	.002
School protective factors	NA	NA	0.9 (0.9-1.0)	<.001	1.0 (1.0-1.0)	.04
Family conflict	NA	NA	1.1 (1.1-1.2)	<.001	1.1 (1.0-1.2)	.002
Racial and ethnic discrimination	NA	NA	1.2 (1.0-1.5)	.05	0.9 (0.7-1.2)	.43
BPM questionnaire score						
Internalizing	NA	NA	NA	NA	1.1 (1.1-1.1)	<.001
Externalizing	NA	NA	NA	NA	1.0 (1.0-1.0)	.10
CBCL questionnaire score						
Internalizing	NA	NA	NA	NA	1.0 (1.0-1.0)	.01
Externalizing	NA	NA	NA	NA	1.0 (1.0-1.0)	.004

Abbreviations: BPM, Brief Problem Monitor; CBCL, Child Behavioral Checklist; NA, not applicable; OR, odds ratio.

^a^ Binary logistic regression models were used, with cyberbullying experiences and experiences or perpetration of offline peer aggression as the independent variables and suicidality as the dependent variable.

^b^ Model 1 covaries for age, sex, race (Black or White), ethnicity (Hispanic), and parent education.

^c^ Model 2 covaries as in model 1 plus negative life events, parental monitoring, school protective factors, family conflict, and scores on a 7-item racial and ethnic discrimination measure Adolescent Brain Cognitive Development study variable name dim_y_ss_mean.

^d^ Model 3 includes all covariates from model 2 plus psychopathology measures (parent and child reports of internalizing and externalizing symptoms on the CBCL and BPM questionnaires, respectively).

^e^ Experiences or perpetration of offline peer aggression were defined as scoring in the top decile range on the summary measure of each offline peer experience measure (overt aggression, relational aggression, and reputational aggression).

### Sensitivity Analyses

Results from the main analyses remained similar when using different measures of cyberbullying and offline peer aggression experiences and suicidality for the sensitivity analyses. Analyses that included the indication of a power imbalance (endorsed by 273 participants [2.6% of the entire cohort]) showed an association of experiencing cyberbullying with power imbalance with suicidality when accounting for demographics and environmental factors. However, this association was no longer present when accounting for psychopathology. Cyberbullying with a power imbalance was endorsed by only 29 participants (0.3%) and was associated with suicidality accounting for demographics; but this association was not statistically significant when covarying for environmental factors or psychopathology (eTable 2 in the Supplement).

Analyses including indicators of chronicity of cyberbullying involvement revealed that among 613 participants (5.9% of the entire cohort) who endorsed past-year experiences of cyberbullying, there was a dose-response association of cyberbullying frequency and suicidality that remained significant when accounting for demographics, environmental factors, and psychopathology. Among participants reporting past-year cyberbullying perpetration frequency, association with suicidality was significant when covarying for demographics but not when further covarying for environmental factors and psychopathology (eTable 3 in the Supplement).

Results of the evaluation of each individual component of the offline experiences or perpetration of peer aggression measures (overt, relational, and reputational) instead of summary measures were similar to the main analyses (eTable 4 in the Supplement). Sensitivity analyses testing the association of cyberbullying experiences and suicidality revealed results similar to those of the main analyses when accounting for site and family relatedness and when assessing suicidal ideation and attempts separately (eTables 5 and 6 in the Supplement, respectively).

## Discussion

In this large cross-sectional study of US adolescents, cyberbullying experiences were relatively prevalent (9.0% of the cohort) and were associated with suicidality (ideation or attempts) over and above multiple confounders, including offline peer aggression. In contrast, cyberbullying perpetration was less prevalent (<1.0%), was highly correlated with being a cyberbullying target (2 of 3 perpetrators of cyberbullying were also targets), and was not independently associated with suicidality. Because the prevalence of cyberbullying experiences in the ABCD study is similar to recent reports^61,62,63^ and because this study accounted for multiple environmental risk and protective factors (including offline peer aggression), our findings point to cyberbullying experiences as an independent risk factor for youth suicidality. We therefore suggest that this study can inform clinical risk stratification and youth suicide prevention initiatives.

Our findings provide 3 clinically informative insights on (1) the differences between experiencing and perpetrating cyberbullying compared with offline peer aggression and (2) their distinct associations with youth suicidality. First, in contrast with previous studies,^33,34,35,64,65^ we found that cyberbullying experiences only partly overlap with offline peer aggression experiences, with most targets of cyberbullying not reporting being targets or perpetrators of offline peer aggression. This finding supports the notion that cyberbullying is a distinct phenomenon, independent of offline peer aggression experiences,^24,31,32,66^ and suggests that (1) adolescents affected by cyberbullying are different from those affected by offline peer aggression and (2) screening for cyberbullying experiences may detect youths at risk who are not detected when screening for offline peer aggression experiences. Second, we report a difference between experiencing and perpetrating cyberbullying in terms of their relationship with suicidality, whereas experiencing and perpetrating offline peer aggression were both associated with suicidality. Third, we report that cyberbullying experiences seem to be an independent stressor associated with adolescent suicidality, even when accounting for offline peer aggression experiences.

Our results contradict previous research suggesting that perpetrators of cyberbullying are at increased risk for suicidality, more so than perpetrators of offline peer aggression.^25,26^ However, these studies acknowledge that cyberbullying is a more recent phenomenon that requires further investigation.^25,26^ Thus, a few potential explanations may account for the fact that cyberbullying is not associated with suicidality in this sample but offline peer aggression is. First, it has been suggested that perpetrator anonymity may be key to cyberbullying behavior.^67,68,69^ Perpetrator anonymity may lead to lower levels of distress for the perpetrator and thus a lesser mental health burden than offline peer aggression, as perpetrators of cyberbullying are often unaware of the distress they cause the target and do not fear punishment for their behavior.^70,71,72^ Furthermore, adolescents tend to respond to any online peer interaction automatically, without giving much thought to their actions.^71^ Indeed, the majority of cyberbullying occurs on instant messaging platforms.^69,71^ Adolescents may therefore be quicker to engage in cyberbullying and quickly pull the “cyberbullying trigger” without fully understanding the magnitude of their actions or viewing them as offensive.^72^ Furthermore, considering evidence from prospective studies showing high psychopathology in perpetrators of offline peer aggression even before they express peer aggression,^73^ it is likely that they are developmentally more prone to suicidality but data on longitudinal trajectories of cyberbullying are limited. Finally, we cannot rule out measurement bias, with evidence suggesting that cyberbullying may be more difficult to accurately determine compared with experiences or perpetration of offline peer aggression.^10^

This study has some immediate implications. For clinicians working directly with adolescents, this work suggests that cyberbullying experiences are associated with suicidality over and above multiple known risk factors; therefore, it may be prudent to ask adolescents about this exposure as part of primary care evaluations.^74^ For researchers, our cross-sectional findings on the specificity of the association of cyberbullying experiences and adolescent suicidality should propel replication and further longitudinal analyses aiming to dissect the causal link between cyberbullying and suicidality and to delineate potential mechanisms underlying this link. Notably, although the current analysis cannot establish causality, we did find a dose-response association of frequency of past-year cyberbullying experiences and suicidality even when accounting for multiple confounders and psychopathology. For policy makers wishing to optimize youth suicide prevention efforts, this study should further encourage addressing of cyberbullying experiences in interventions.^75,76^

### Limitations

A few methodologic limitations should be considered when interpreting our findings. First, the cross-sectional nature of the study precludes causal inference. Second, because of its large size and comprehensive broad phenotyping, the ABCD study used a low-resolution screening measure of cyberbullying experiences. Future focused studies should include more thorough and in-depth measurement of cyberbullying characteristics, their subtypes, and their associations with suicidality. Third, although we controlled for multiple known stressors and protective factors previously associated with suicidality in the ABCD study, the role of unmeasured confounders cannot be discounted. This is highlighted by continuous attenuation of associations with adjustment for more covariates in all models, which may suggest residual confounding. Fourth, although the ABCD study is the largest youth sample to phenotype for experiences or perpetration of cyberbullying, offline peer aggression, and suicidality, the number of participants who endorsed perpetration of cyberbullying, and specifically who endorsed perpetration but not experiencing cyberbullying, was relatively low compared with those reporting cyberbullying experiences (96 and 30 vs 930, respectively), which may have reduced power. Finally, although this study leveraged data collected recently (from mid-2018 to early 2021), most participants (72.0%) were evaluated before the COVID-19 pandemic.^77^ Indeed, future research on youth suicidality is needed to determine the significance of changes in peer online communication^14,15,16^ and cyberbullying during the COVID-19 pandemic.^12^

## Conclusions

The findings of this study suggest that experiencing, but not perpetrating, cyberbullying is associated with adolescent suicidality above and beyond other forms of peer aggression experiences and established risk and protective factors. Assessment of cyberbullying experiences among children and adolescents should be a component of the comprehensive suicide risk assessment.

## Notes

Supplement. **eTable 1.** Overlap of Cyberbullying and Offline Peer Aggression Experiences **eTable 2.** Association of Cyberbullying Experiences With Indicators of Power Imbalance and Suicidality **eTable 3.** Association of Frequency of Cyberbullying Experiences and Suicidality **eTable 4.** Association of Different Types of Offline Peer Aggression Experiences and Suicidality **eTable 5.** Association of Cyberbullying Experiences and Suicidality Accounting for Site and Family Relatedness **eTable 6.** Association of Cyberbullying Experiences and Suicidal Ideation and Suicide Attempts **eFigure.** Correlation Matrix Describing Associations of Offline Peer Aggression Experiences
