# Supplementary material for: Association of Cyberbullying Experiences and Perpetration With Suicidality in Early Adolescence
Source: JAMA Netw Open. 2022 Jun 27;5(6):e2218746. doi: 10.1001/jamanetworkopen.2022.18746 (PMC9237787; doi:10.1001/jamanetworkopen.2022.18746)
Supplement: Supplement. — eTable 1. Overlap of Cyberbullying and Offline Peer Aggression Experiences eTable 2. Association of Cyberbullying Experiences With Indicators of Power Imbalance and Suicidality eTable 3. Association of Frequency of Cyberbullying Experiences and Suicidality eTable 4. Association of Different Types of Offline Peer Aggression Experiences and Suicidality eTable 5. Association of Cyberbullying Experiences and Suicidality Accounting for Site and Family Relatedness eTable 6. Association of Cyberbullying Experiences and Suicidal Ideation and Suicide Attempts eFigure. Correlation Matrix Describing Associations of Offline Peer Aggression Experiences [file jamanetwopen-e2218746-s001.pdf]

Supplementary Online Content

Arnon S, Brunstein Klomek A, Visoki E, et al. Association of cyberbullying experiences and perpetration with suicidality in early adolescence. *JAMA Netw Open*. 2022;5(6):e2218746. doi:10.1001/jamanetworkopen.2022.18746

eTable 1. Overlap of Cyberbullying and Offline Peer Aggression Experiences

eTable 2. Association of Cyberbullying Experiences With Indicators of Power Imbalance and Suicidality

eTable 3. Association of Frequency of Cyberbullying Experiences and Suicidality

eTable 4. Association of Different Types of Offline Peer Aggression Experiences and Suicidality

eTable 5. Association of Cyberbullying Experiences and Suicidality Accounting for Site and Family Relatedness

eTable 6. Association of Cyberbullying Experiences and Suicidal Ideation and Suicide Attempts

eFigure. Correlation Matrix Describing Associations of Offline Peer Aggression Experiences

This supplementary material has been provided by the authors to give readers additional information about their work.

© 2022 Arnon S et al. *JAMA Network Open*.

eTable 1. Overlap of Cyberbullying and Offline Peer Aggression Experiences

		**Targets of cyberbullying (N=930)**	
		n	%
**Target of high levels of offline peer aggression**	Yes	262	28.2%
	No	668	71.8%
**Perpetrator of high levels of offline peer aggression**	Yes	182	19.6%
	No	748	80.4%

High offline peer aggression was determined for participants who scored at the top decile of the summary measure of offline peer aggression experiences (overt aggression, relational, and reputational).

© 2022 Arnon S et al. *JAMA Network Open*.

eTable 2. Association of Cyberbullying Experiences With Indicators of Power Imbalance and Suicidality

	Model 1[a]		Model 2[b]		Model 3[c]	
	**OR (95% CI)**	**P**	**OR (95% CI)**	**P**	**OR (95% CI)**	**P**
**Target of cyberbullying**	5.6 (4.2-7.4)	<.001	2.5 (1.8-3.5)	<.001	1.4 (0.9-2.2)	0.10
**Perpetrator of cyberbullying**	3.2 (1.2-8.4)	0.02	0.8 (0.2-2.8)	0.73	1.3 (0.3-4.9)	0.74
**Negative life events**			1.2 (1.1-1.2)	<.001	1.1 (1.1-1.1)	<.001
**Parental monitoring**			0.6 (0.5-0.7)	<.001	0.7 (0.6-0.9)	0.002
**School protective factors**			0.9 (0.9-1.0)	<.001	1.0 (1.0-1.0)	0.03
**Family conflict**			1.2 (1.1-1.2)	<.001	1.1 (1.0-1.2)	<.001
**Racial/ethnic discrimination**			1.5 (1.2-1.8)	<.001	1.0 (0.7-1.3)	0.88
**BPM externalizing score**					1.0 (1.0-1.0)	0.03
**BPM internalizing score**					1.1 (1.1-1.1)	<.001
**CBCL internalizing score**					1.0 (1.0-1.0)	0.03
**CBCL externalizing score**					1.0 (1.0-1.0)	0.002

Binary logistic regression models of cyberbullying experiences (being a target of) with power imbalance (endorsed by n=273) and cyber-bullying perpetration with power imbalance (endorsed by n=30) as the independent variables; and suicidality as the dependent variable.

[a]Model 1 covaries for age, gender, race (White, Black), Hispanic ethnicity, parent education.

[b]Model 2 covaries as above + bad life events, parent monitoring, school protective factors, family conflict, 7-item racial/ethnic discrimination measure.

[c]Model 3 includes all covariates from Model 2 + psychopathology measures (parent and youth report, internalizing and externalizing symptoms).

Abbreviations: OR, odds ratio; 95% CI, 95% confidence interval.

© 2022 Arnon S et al. *JAMA Network Open*.

eTable 3. Association of Frequency of Cyberbullying Experiences and Suicidality

	Model 1[a]		Model 2[b]		Model 3[c]	
	**OR (95% CI)**	**P**	**OR (95% CI)**	**P**	**OR (95% CI)**	**P**
**Target of cyberbullying[d]**	1.6 (1.5-1.7)	<.001	1.3 (1.3-1.5)	<.001	1.3 (1.1-1.4)	<.001
**Perpetrator of cyberbullying[d]**	0.9 (0.7-1.1)	0.26	0.7 (0.5-1.0)	0.03	0.5 (0.3-0.9)	0.02
**Negative life events**			1.2 (1.1-1.2)	<.001	1.1 (1.0-1.1)	<.001
**Parental monitoring**			0.6 (0.5-0.7)	<.001	0.7 (0.6-0.9)	0.002
**School protective factors**			0.9 (0.9-1.0)	<.001	1.0 (1.0-1.0)	0.05
**Family conflict**			1.2 (1.1-1.2)	<.001	1.1 (1.1-1.2)	<.001
**Racial/ethnic discrimination**			1.5 (1.2-1.8)	<.001	1.0 (0.7-1.3)	0.79
**BPM externalizing score**					1.0 (1.0-1.0)	0.02
**BPM internalizing score**					1.1 (1.1-1.1)	<.001
**CBCL internalizing score**					1.0 (1.0-1.0)	0.03
**CBCL externalizing score**					1.0 (1.0-1.0)	0.006
	**OR (95% CI)**	**P**	**OR (95% CI)**	**P**	**OR (95% CI)**	**P**
**Target of cyberbullying[e]**	1.6 (1.5-1.7)	<.001	1.3 (1.2-1.4)	<.001	1.2 (1.1-1.4)	<.001
	**OR (95% CI)**	**P**	**OR (95% CI)**	**P**	**OR (95% CI)**	**P**
**Perpetrator of cyberbullying[e]**	1.5 (1.2-1.9)	<.001	1.0 (0.8-1.3)	0.94	0.7 (0.4-1.1)	0.12

Binary logistic regression models with past year frequency of being a target of cyberbullying (reported by 613 participants) and perpetrator of cyberbullying (reported by 56 participants) as the independent variables; and suicidality as the dependent variable. Frequency was measured on a 1-8 scale (1=1 time; 2=2 times; 3=3 times; 4=4-9 times; 5=10-19 times; 6=20-39 times; 7=40-49 times; 8=50+ times). Participants that reported they had no experiences as targets or perpetrators of cyberbullying were coded as 0.

[a]Model 1 covaries for age, gender, race (White, Black), Hispanic ethnicity, parent education.

[b]Model 2 covaries as above + bad life events, parent monitoring, school protective factors, family conflict, 7-item racial/ethnic discrimination measure.

[c]Model 3 includes all covariates from Model 2 + psychopathology measures (parent and youth report, internalizing and externalizing symptoms).

[d]Results derived from models that include both frequencies of being a target and perpetrator of cyberbullying as the independent variables.

[e]Results derived from models that include frequency of only one cyber-exposure at a time, either being a target of cyberbullying or a perpetrator of cyberbullying frequency (and not both as in models above). These models were estimated due to the flip in the direction of association between cyber-bullying and suicidality obtained in the model that included both cyber-exposures frequency. This flip in direction points to co-linearity between independent variables (i.e., frequency of being a target and a perpetrator of cyberbullying) and is addressed by running models that include only being a target or perpetrator separately, as done here.

Abbreviations: OR, odds ratio; 95% CI, 95% confidence interval; BPM, Brief Problem Monitor questionnaire; CBCL, Child Behavioral Checklist questionnaire.

© 2022 Arnon S et al. *JAMA Network Open*.

eTable 4. Association of Different Types of Offline Peer Aggression Experiences and Suicidality

		Model 1[a]		Model 2[b]		Model 3[c]	
**Type of offline aggression experiences**	**Role**	**OR (95% CI)**	**P**	**OR (95% CI)**	**P**	**OR (95% CI)**	**P**
**Offline overt aggression**	Target	1.3 (1.3-1.4)	<.001	1.2 (1.1-1.3)	<.001	1.1 (1.1-1.2)	<.001
	Perpetrator	1.3 (1.2-1.4)	<.001	1.1 (1.1-1.2)	<.001	1.1 (1.0-1.2)	0.11
**Offline relational aggression**	Target	1.3 (1.2-1.3)	<.001	1.2 (1.2-1.2)	<.001	1.1 (1.1-1.2)	<.001
	Perpetrator	1.2 (1.1-1.3)	<.001	1.1 (1.0-1.1)	0.006	1.1 (1.0-1.1)	0.09
**Offline reputational aggression**	Target	1.3 (1.3-1.3)	<.001	1.2 (1.1-1.2)	<.001	1.1 (1.0-1.1)	<.001
	Perpetrator	1.2 (1.1-1.3)	<.001	1.1 (1.0-1.2)	0.05	1.1 (1.0-1.2)	0.12

Binary logistic regression models with three different types of offline peer aggression experiences as the independent variables; and suicidality as the dependent variable. Each panel represents a separate model that includes both the target and the perpetrator role as independent variables.

[a]Model 1 covaries for age, gender, race (White, Black), Hispanic ethnicity, parent education.

[b]Model 2 covaries as above + bad life events, parent monitoring, school protective factors, family conflict, 7-item racial/ethnic discrimination measure.

[c]Model 3 includes all covariates from Model 2 + psychopathology measures (parent and youth report, internalizing and externalizing symptoms).

[d]Defined as scoring on the top decile range in the summary measure of each of the offline peer aggression experiences measures (overt aggression, relational, and reputational peer aggression).

Abbreviations: OR, odds ratio; 95% CI, 95% confidence interval.

© 2022 Arnon S et al. *JAMA Network Open*.

eTable 5. Association of Cyberbullying Experiences and Suicidality Accounting for Site and Family Relatedness

	Model 1[a]		Model 2[b]		Model 3[c]	
	**OR (95% CI)**	**P**	**OR (95% CI)**	**P**	**OR (95% CI)**	**P**
**Target of cyberbullying**	4.3 (3.5-5.1)	<.001	2.5 (2.0-3.1)	<.001	1.8 (1.4-2.4)	0.002
**Perpetrator of cyberbullying**	1.4 (0.8-2.4)	0.38	0.7 (0.4-1.4)	0.20	0.6 (0.3-1.4)	0.17
**Negative life events**			1.2 (1.1-1.2)	<.001	1.1 (1.0-1.1)	<.001
**Parental monitoring**			0.6 (0.5-0.7)	<.001	0.7 (0.6-0.9)	<.001
**School protective factors**			0.9 (0.9-1.0)	<.001	1.0 (1.0-1.0)	0.03
**Family conflict**			1.2 (1.1-1.2)	<.001	1.1 (1.0-1.2)	0.001
**Racial/ethnic discrimination**			1.4 (1.2-1.7)	0.003	1.0 (0.7-1.3)	0.80
**BPM externalizing score**					1.0 (1.0-1.0)	0.02
**BPM internalizing score**					1.1 (1.1-1.1)	<.001
**CBCL internalizing score**					1.0 (1.0-1.0)	0.05
**CBCL externalizing score**					1.0 (1.0-1.0)	0.003

[a]Model 1 covaries for age, gender, race (White, Black), Hispanic ethnicity, parent education.

[b]Model 2 covaries as above + bad life events, parent monitoring, school protective factors, family conflict, 7-item racial/ethnic discrimination measure.

[c]Model 3 includes all covariates from Model 2 + psychopathology measures (parent and youth report, internalizing and externalizing symptoms).

Abbreviations: OR, odds ratio; 95% CI, 95% confidence interval; BPM, Brief Problem Monitor questionnaire; CBCL, Child Behavioral Checklist questionnaire.

© 2022 Arnon S et al. *JAMA Network Open*.

eTable 6. Association of Cyberbullying Experiences and Suicidal Ideation and Suicide Attempts

	Model 1[a]		Model 2[b]		Model 3[c]	
Dependent variable: Suicidal ideation	**OR (95% CI)**	**P**	**OR (95% CI)**	**P**	**OR (95% CI)**	**P**
**Target of cyberbullying**	4.2 (3.5-5.1)	<.001	2.5 (2.0-3.1)	<.001	1.8 (1.4-2.4)	<.001
**Perpetrator of cyberbullying**	1.4 (0.8-2.4)	0.28	0.7 (0.4-1.3)	0.27	0.6 (0.3-1.4)	0.27
Dependent variable: Suicide attempt	**OR (95% CI)**	**P**	**OR (95% CI)**	**P**	**OR (95% CI)**	**P**
**Target of cyberbullying**	6.1 (4.2-8.8)	<.001	2.9 (2.0-4.4)	<.001	2.7 (1.6-4.4)	<.001
**Perpetrator of cyberbullying**	1.3 (0.5-3.5)	0.54	0.7 (0.2-2.0)	0.48	0.3 (0.1-1.4)	0.14

Binary logistic regression models with being a target and perpetrator of cyberbullying as the independent variables; and suicidality as the dependent variable. Each panel represents separate models that include different dependent variables (suicidal ideation or suicide attempts).

[a]Model 1 covaries for age, gender, race (White, Black), Hispanic ethnicity, parent education.

[b]Model 2 covaries as above + bad life events, parent monitoring, school protective factors, family conflict, 7-item racial/ethnic discrimination measure.

[c]Model 3 includes all covariates from Model 2 + psychopathology measures (parent and youth report, internalizing and externalizing symptoms).

Abbreviations: OR, odds ratio; 95% CI, 95% confidence interval.

© 2022 Arnon S et al. *JAMA Network Open*.

eFigure. Correlation Matrix Describing Associations of Offline Peer Aggression Experiences

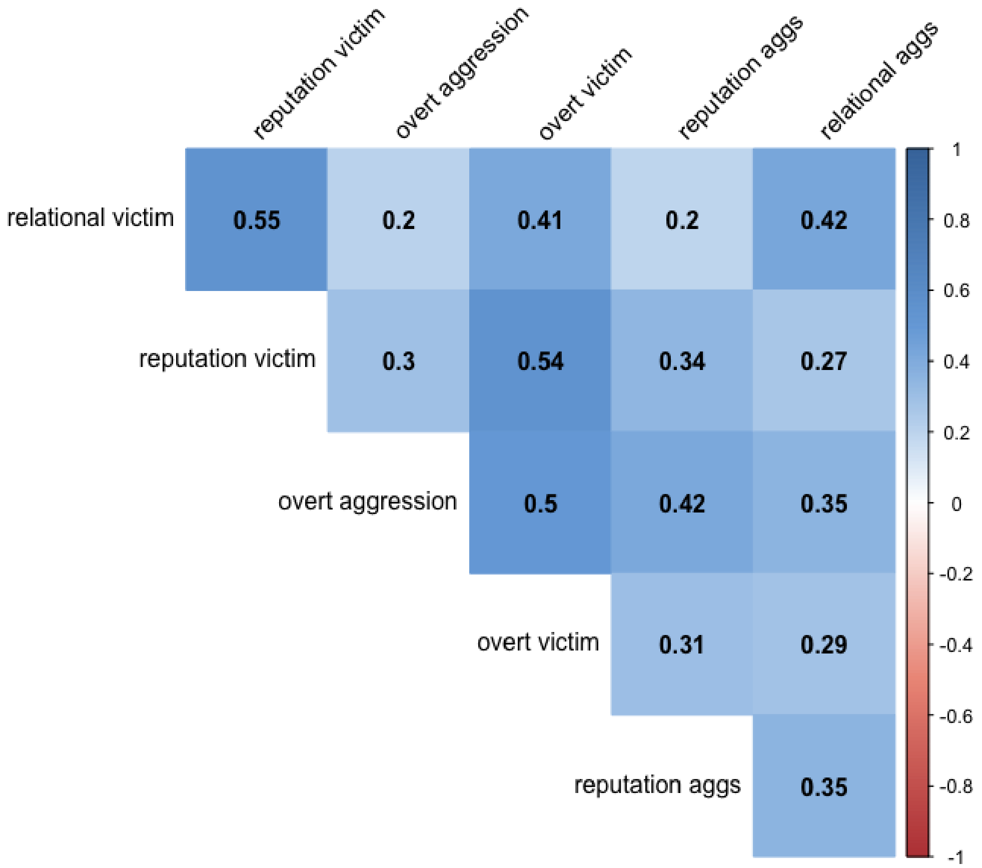

© 2022 Arnon S et al. *JAMA Network Open*.
